# Antimicrobial activity of vinegar water in chilled chicken carcasses

**DOI:** 10.1016/j.psj.2026.106674

**Published:** 2026-02-19

**Authors:** Carly Long, Md Shafiul Islam Rion, Corey Coe, Claire Suszynski, Reuben Adejumo, Joe Moritz, Annette Freshour, Cassandra Orndorff, Timothy Boltz, Lisa Jones, Cangliang Shen

**Affiliations:** a School of Agriculture and Food System, West Virginia University, Morgantown, WV 26506, USA; b Department of Poultry Science, Mississippi State University, Mississippi State, MS 39762, USA

**Keywords:** Campylobacter, Chilling, Enterococcus faecium, Salmonella, Vinegar water

## Abstract

Very small poultry producers in West Virginia are very interested in learning the antimicrobial activities of commercial vinegar water during post-harvest broiler processing. We aimed to 1) evaluate the efficacy of vinegar water against pathogens and surrogate bacteria during the chilling of broiler carcasses, and 2) evaluate the anti-bacterial efficacy of 0, 50, and 75% vinegar water against surrogate bacteria during chilling at the Mobile Poultry Processing Unit (**MPPU**) pilot plant facility. In Study I, fresh organic broiler carcasses were inoculated with *Salmonella* Typhimurium, *Campylobacter jejuni*, and *Enterococcus faecium,* followed by chilling in the refrigerated 0, 50%, and 75% vinegar water for 1 or 24 h. In Study II, the MPPU-processed fresh broiler carcasses were chilled for 24 h in 0, 50, and 75% vinegar, 5 ppm of chlorine, and 2.5% of lactic/citric acid blend solutions. The broiler carcasses were then processed with D/E neutralizing solution in a standard poultry sampling bag for 30 seconds, followed by spread-plating onto tryptic soy agar plus 200 ppm-nalidixic acid (**TSA-NaL**), Brucella, and Bile-esculin agars (**BEA**)-NaL for *S.* Typhimurium, *C. jejuni*, and *E. faecium*, respectively. TSA- and BEA-NaL were incubated at 35°C for 48 h, and the Brucella agars were incubated in a microaerophilic jar at 42°C for 48 h. Mixed model procedure in R-program (3 × 2 × 3 factorial design) was used for data analysis with a significance level of *P*=0.05. Results showed that chilling in 50 and 75% of vinegar water for 1 h reduced (*P* < 0.05) 0.76-1.85 and 1.46-2.00 log_10_ CFU/mL of *S*. Typhimurium and *C. jejuni*, and when the chilling time was extended to 24 h, the reductions increased to 1.20-2.52 and 1.8-2.27 log_10_ CFU/mL, respectively. *E. faecium* showed less reduction (0.2-0.7 log_10_CFU/mL, *P* < 0.05) than *S.* Typhimurium. MPPU pilot study showed that chilling in 50 and 75% vinegar water for 24 h reduced (*P* < 0.05) *E. faecium* by 3.29-3.64 log_10_CFU/mL. Results suggested that applying 50 or 75% of commercial vinegar water for chilling 24h is effective to control foodborne pathogens on broiler carcasses, and *E. faecium* is a promising *Salmonella* surrogate during post-harvest validation studies.

## Introduction

In the United States, chicken is the primary animal protein consumed (U.S. Department of Agriculture Economic Research Service, 2022), and the annual consumption of chicken per person increased from 22.4 pounds in 1970 to 54.6 pounds per person per year in 2019 (U.S. Department of Agriculture Economic Research Service, 2025). Meanwhile, as the demand for locally produced chicken products increases, pastured poultry production and on-farm mobile poultry harvesting have become more prevalent. The West Virginia Department of Agriculture currently permits small local producers to harvest up to 20,000 birds on farms without inspection each year (West Virginia Department of Agriculture, 2023). In recent years, several Mid-Atlantic states, including Kentucky, Pennsylvania, Ohio, and Massachusetts, have implemented Mobile Poultry Processing Units (**MPPUs**) to provide small-scale poultry producers with accessible, on-farm processing options and reduce their dependence on large commercial facilities (Li, 2017). Foodborne illness remains a significant public health problem in the United States, as it is responsible for over 48 million cases per year and 3,000 deaths (Centers for Disease Control and Prevention (CDC), 2025). Bacterial contamination resulting from improper food processing, which can lead to microbial cross-contamination, remains the primary cause of foodborne illness in the United States. Among the pathogens responsible, *Salmonella* and *Campylobacter* infections are the most prevalent. Between 1998 and 2022, whole chicken carcasses were linked to 15 major outbreaks, resulting in a total of 896 illnesses and 231 hospitalizations (Chard et al., 2025). These bacteria commonly reside in the intestinal tracts of apparently healthy chickens, allowing contamination to occur during slaughter and processing even when birds show no visible signs of disease (Thames and Sukumaran, 2020). There are concerns that poultry processed in MPPUs and sold at local farmers’ markets may elevate food microbial safety risks. Recent studies have reported the presence of both *Salmonella* and *Campylobacter* after scalding, eviscerating, and water chilling, highlighting the importance of adding antimicrobials to the chilling water (Stearns et al., 2024). With the rising popularity of locally raised poultry, assessing microbial hazards and developing strategies to reduce them is increasingly important.

### Significance of the study

To address the multiple processing procedures where broilers may be contaminated with foodborne pathogens, multiple antimicrobial controls are applied at various steps to create a multi-hurdle approach. Commercial antimicrobials are used, including inside-outside bird washes (**IOBW**), chiller applications, and post-chill applications. Acetic acid (≤ 5%), sodium hypochlorite (referred as free chlorine, ≤ 50 ppm), and a lactic and citric acid blend (≤ 2.5%), have been approved by the U.S. Department of Agriculture – Food Safety and Inspection Services (**USDA-FSIS**) to inhibit foodborne pathogens during poultry meat processing (United States Department of Agriculture-Food Safety and Inspection Service, 2021). In the U.S., poultry chilling is an immersion chill process where antimicrobials can be added. Historically, 50 ppm of chlorine, 85 ppm of peroxyacetic acid, and 2.5% of lactic and citric acids have been used in chillers (McKee, 2012). Studies have been well documented that chillers with antimicrobials can significantly reduce pathogens and microbial cross-contamination on broiler carcasses if operating properly (Russell, 2012). Previous research has demonstrated that water alone is not effective in reducing microbial growth after the inoculation of non-native bacteria (Nagel et al., 2013). Specifically, for MPPU, the chiller is the only place that can be used for adding antimicrobials. With the growing market demand for “clean label” meat products, conventional antimicrobials such as chlorine are often perceived as problematic due to its chemical nature, such as the generation of chlorine byproducts (Shen et al., 2016). This concern increases interest in alternative interventions such as applying vinegar-based treatments. Given that the optimum pH range for *Salmonella* Typhimurium, *Campylobacter jejuni*, and *Enterococcus faecium* is 6.5-7.5, it is expected that the acidic conditions of the treatment (2.5-2.9) will demonstrate effective antimicrobial activity against the pathogens especially with the present 5% acetic acid. Poultry processing plants do not typically introduce biosafety level 2 pathogens into their Hazard Analysis and Critical Control Point (**HACCP**) systems, to determine Critical Control Points (**CCPs**) due to associated biosafety risks and practical constraints. Our previous studies have utilized *Enterococcus faecium* as a *Salmonella* surrogate for pelleting experiments (Boney et al., 2018) and both standard and aggressive thermal pelleting of feed (Boltz et al., 2019). The behavior of *E. faecium* in the chilling process also needs to be validated in both laboratory and pilot plant conditions.

### Aim of the study

We aimed to 1) evaluate the antimicrobial efficacy of using commercial distilled vinegar water in the chiller against *S.* Typhimurium, *C. jejuni*, and *E. faecium* on broiler carcasses; and 2) validate the antimicrobial efficacy of vinegar water in the chiller against *E. faecium* on broiler carcasses processed in the MPPU at the West Virginia University poultry farm.

## Materials and methods

### Institutional ethical approval number and statement

All animals (live birds) were reared according to protocols approved by the West Virginia University Animal Care and Use Committee (IACUC Protocol #1602000612_R2).

### Bacterial strains used in this study

In this study, *S.* Typhimurium ATCC 14028, and its surrogate *E. faecium* ATCC 2625, and two strains of *C. jejuni* RM5032 and RM1188 (donated from Dr. Nereus Gunther from USDA-ARS, Wyndmoor, PA, U.S.A.) were used for laboratory and MPPU pilot plant tests. *S*. Typhimurium and *E. faecium* were activated from -80°C frozen stock cultures and then streak-plated onto tryptic soy agar (**TSA**, Hardy Diagnostics, Santa Maria, CA, U.S.A) and bile esculin agar (**BEA**, Hardy Diagnostics) plus 200 ppm of nalidixic acid (**NaL**, Hardy Diagnostics) plates, respectively, and then incubated at 35°C for 24 to 48 h followed by being stored at 4°C. *C. jejuni* were activated from the frozen culture onto Brucella agar (Hardy Diagnostics), followed by incubating for 48 h and stored in a microaerophilic jar (Hardy Diagnostics) at 4°C. Stock cultures on agar plates were re-streaked onto new agar plates every three weeks during the experimental period.

### Preparation for inoculum

The day before the experiment, two colonies of *S*. Typhimurium and *E. faecium* were picked using a sterile plastic loop from the TSA-NaL and BEA-NaL agars, respectively, and placed into 10 mL of sterilized tryptic soy broth (**TSB**, Hardy Diagnostics) followed by incubating at 35°C for 24 h. For *C. jejuni*, two single colonies from the stock Brucella agars were transferred into 10 mL of Bolton’s broth (Hardy Diagnostics) followed by incubating at 42°C for 48 h in a 2.5 L microaerophilic jar (5.0% O_2_, 10% CO_2_, and 85% N_2_) in a 2.5-L microaerophilic jar with gas generator (Hardy Diagnostics). Triplicate tubes (10 mL of each tube) were prepared for each bacterial strain solution.

Immediately before inoculation, tubes of 24 h bacterial solutions of each individual bacterial culture were centrifuged for 10 min at 6 × 1000 g in a refrigerated centrifuge (VWR Symphony 4417, VWR International, Radnor, PA, U.S.A). The supernatant was discarded, and then the precipitated cells were washed in 10 mL of 0.1% buffered peptone water (**BPW**, Hardy Diagnostics) by centrifuging for an additional 10 min. The final washed and resuspended bacterial solution in each tube was then vortexed for 30 s to thoroughly mix the cells before being combined to form a 30-mL solution. Then, from this mixed solution, 1 mL was added to 9 mL of 0.1% BPW and vortexed well to generate a 10 mL inoculum for chicken carcasses.

### Purchasing and preparation of broiler carcasses for laboratory tests

Whole broiler carcasses were purchased from a retail grocery store in Morgantown, WV, 24 h before the experiment and maintained in a refrigerated cooler at 5 ± 1°C until the experiment was set up. The average weight of the broiler carcasses was 2.5 kg.

### Inoculation of broiler carcasses

Broiler carcasses were removed from refrigeration, unwrapped, and air-dried on foil paper under a biohazard hood for 15 min before inoculation. The inoculation was conducted by pipetting 3 drops of 200 µL of the prepared bacterial inoculum solution on medial side of the carcasses and then flipped over for adding another 3 drops of the 200 µL solution on the lateral sides followed by staying in the biohazard hood for 15 min to allow bacterial attachment. The inoculation level of *S*. Typhimurium, *E. faecium*, and *C. jejuni* were 7.31, 7.24, and 5.64 log_10_ colony-forming units (**CFU**)/mL per carcass rinsate, respectively.

### Preliminary laboratory studies

Before the start of the experimental settings, preliminary studies were conducted to test the efficacy of 4.8, 9.6, 12.5, and 25% vinegar water against *S.* Typhimurium in chilled broiler carcasses for 1 h. The preparation of bacterial inoculum and inoculation of broiler carcasses followed the same procedures as described above. We also randomly picked 6 commercial retail broiler samples using modified Food & Drug Administration- Bacteria Analytical Manual (**FDA-BAM**) protocol in our previous study (Li et al., 2017) to test the presence/absence of natural *Salmonella* and no presumptive *Salmonella* colonies were detected.

### Chilling of broiler carcasses in vinegar water

Inoculated broiler carcasses were immersed for 1 and 24 h in a chilled water tank (68 L) containing 2 kg of ice and the designated vinegar concentrations of 0 (water control, water activity 1.000, pH 5.73), 50 (water activity 0.994, pH 2.47) and 75% (water activity 0.993, pH 1.98). Bottled vinegar water containing 5% acetic acid was pre-stored overnight in the refrigerated cooler and diluted at an appropriate volume to reach the target concentrations. Un-chilled broiler carcasses were also included as controls.

### Poultry farm MPPU pilot studies

The study evaluated 100 birds raised at the West Virginia University (**WVU**) Poultry Farm (Morgantown, WV). All birds were managed in compliance with the WVU Institutional Animal Care and Use Committee guidelines (**IACUC** Protocol #1602000612.1 R2). The flock consisted of day-old male Ross 308 chicks obtained from a commercial hatchery (Myers Poultry, South Fork, PA). Chicks were vaccinated against Marek’s disease and coccidiosis prior to arrival. They were assigned to 56 identical floor pens within 2 rooms (28 pens per room) at a stocking density of 23 birds per pen. Each pen measured 0.8 m × 2.4 m. The rooms under negative pressure and heated with forced air were cross-ventilated. Before chick placement, the rooms were heated to 32°C and gradually decreased (1°C decrease per week) for optimal rearing conditions. The lighting schedule was as follows: 24L:0D from day 0 to day 3, 23L:1D from day 4 to day 7, 20L:4D from day 8 to day 17, 18L:6D from day 18 to day 38. Feed was produced at the West Virginia University pilot feed mill and formulated to contain 56.31% corn, 37.13% soybean meal, 1.25% soybean oil, and provided 20-23% crude protein and 2974 to 3099 kcal/kg of metabolizable energy. Feed and water were administered *as libitum* via feed hoppers and nipple drinkers (2 per pen). Feed was withheld for eight hours before slaughter to standardize gastrointestinal conditions. Fresh pine shavings, evenly spread a day before bird placement, were utilized in the floor pens. Flocks were reared for 38 days in the late spring.

**Fig. 1:**
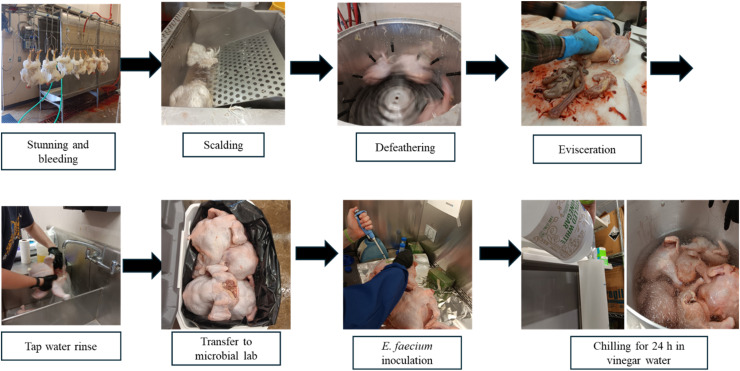
Broiler slaughter process in a mobile poultry processing unit at West Virginia University Poultry Farm.

On day 38, birds were processed in the meat processing laboratory, an enclosed MPPU facility, which consisted of a kill line conveyor, scalder, plucker, stainless steel table (used for manual evisceration), 2-compartment sink, and walk-in refrigerator. A stun knife was used, and a V jugularis cut was made after the broilers were hung on shackles. The broilers were then allowed to bleed out for 5 minutes before being put into the scalder. The broiler processing occurred in the following order: 1) exsanguination for 60 s; 2) scalding in the scalder (Brower, IA, U.S.A) for 60 s at 62.8°C; 3) de-feathering (Ashley Machine Inc., IN, U.S.A); 4) manual evisceration; and 5) carcass chilling at 1.7-2.8°C for 24 h (Fig. 1). The chilling methods used in this study were ice water with the addition of water only, 5 ppm of chlorine solution (sodium hypochlorite, water activity 0.996, pH 5.78), 2.5% lactic and citric acid blend (Chicxide®, Birko, CO, U.S.A, water activity 0.994, pH 1.99), 50 and 75% of distilled vinegar water. The ice water bath was prepared in a 76-L container filled with 18 L of distilled water and ice. The chilling tanks were placed in a walk-in cooler for 24 h. The average temperature of the chilling tanks after 24 h was 4.2 to 4.5°C. Prior to chilling immersion, chicken samples were rinsed under running water to remove visible debris.

### Microbial analysis of chilled broiler carcasses

After chilling, broiler carcasses were immediately added to a pre-sterile poultry sampling bag containing 200 mL of D/E neutralizing solution with hand massage for 30 s to allow even exposure of the carcass to the D/E solution. Then the bags were vigorously shaken for 60 s to detach bacterial cells into the solution. Rinsing solutions were then serially diluted with 9.0 or 9.9 mL of 0.1% BPW, followed by spreading plating onto TSA-NaL, BEA-NaL, and Brucella agars for *S*. Typhimurium, *E. faecium*, and *C. jejuni*, respectively. Agar plates of TSA-NaL and BEA-NaL were then incubated at 35°C for 24 and 48 h, respectively. The Brucella agar plates were incubated for 48 h at 42°C in a microaerophilic jar. After incubation, all plates were manually counted, as recorded by CFU. Three colonies were randomly picked from the Brucella agar for a *C. jejuni* confirmation test using the Campy-latex Agglutination kit (Hardy Diagnostics).

### Data analysis

This study had a 3 × 3 × 2 factorial designs where the reduction of 3 different pathogenic bacteria (*S.* Typhimurium, *E. faecium*, and *C. jejuni*) due to immersing in 3 different solutions (0% vinegar or water control, 50% vinegar, and 75% vinegar) separately for 2 different time periods (1 h and 24 h) were investigated. Bacterial cell number reductions were calculated by subtracting the log-reduction (CFU/mL) value of treatments from the log-reduction (CFU/mL) value of untreated broiler carcasses. A mixed model procedure in R software (version 4.4.1; R Core Team, 2024) was used in the microbial reduction and survival data analysis including individual factors of vinegar concentrations (0, 50%, and 75%), chilling time (1 and 24h) and the interactions between concentrations × chilling time, with significant level at *P* = 0.05. Then, least-square means were calculated by adopting the *emmeans* package (Lenth, 2024) in R (version 4.4.1; R Core Team, 2024) to Figure. out the overall reduction of pathogenic bacteria over the two study periods for all the treatments with a significance level of *P* = 0.05.

## Results

### Preliminary laboratory studies

Immersing broiler carcasses in chilling water containing 4.8, 9.6, 12.5, and 25% vinegar water for 1 h reduced the *S.* Typhimurium cell counts, which ranged from 0.1 to 0.4 log**_10_** CFU/mL.

### Overall results from laboratory studies

**Fig. 2:**
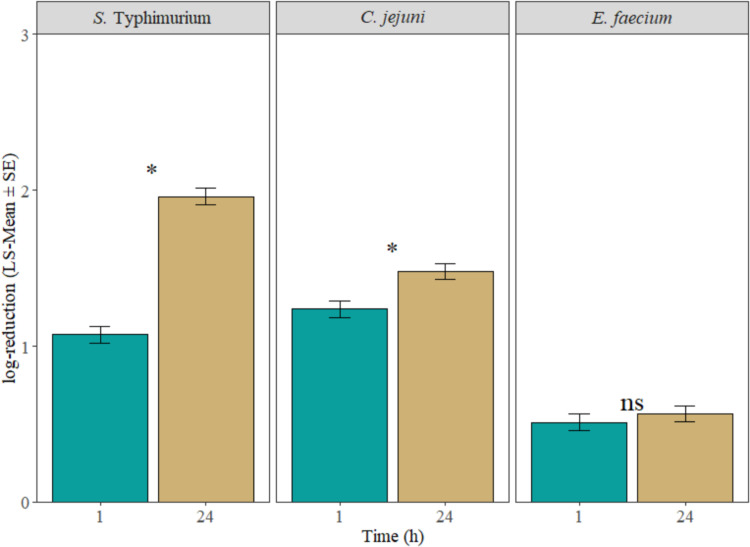
Comparison between 1 and 24 h chilling time for the reductions of Salmonella Typhimurium, Campylobacter jejuni, and surrogate bacteria Enterococcus faecium on broiler carcasses (LS-Mean ± S.E.), regardless of vinegar water concentrations. “*” represent significant difference (P < 0.05).

**Fig. 3:**
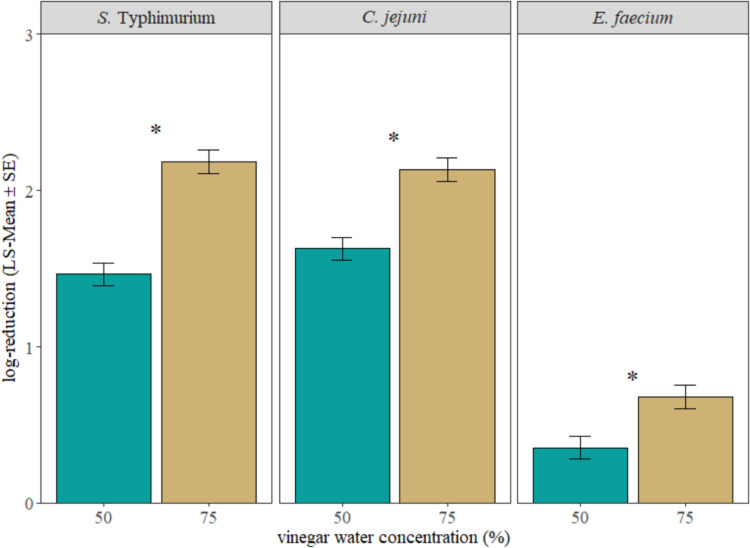
Comparison between 50 and 75% vinegar water chilling for the reductions of Salmonella Typhimurium, Campylobacter jejuni, and surrogate bacteria Enterococcus faecium on broiler carcasses (LS-Mean ± SE), regardless of chilling times. “*” represent significant difference (P < 0.05).

Overall, for *S.* Typhimurium and *C. jejuni*, the LsMeans of chilling broiler carcasses in vinegar water for 24 h increased (*P* < 0.05) the bacterial reductions by 0.88 and 0.24 log_10_ CFU/mL, respectively, compared to the samples chilled for only 1 h (Fig. 2). However, there is no significant difference (*P* > 0.05) in reduction for *E. faecium* between the 1 and 24 h chilling time (Fig. 2). Compared to the 50% vinegar water, applying 75% vinegar water during chilling significantly increased (*P* < 0.05) the bacterial cell count reductions (Fig. 3).

### Results from laboratory studies

The initial cell counts of *S.* Typhimurium on inoculated control broiler carcasses are 7.31± 0.40 log_10_ CFU/mL. After chilling in water (0% vinegar water), 50%, and 75% vinegar water for 1 h, the pathogen population decreased (*P* < 0.05, Table 1). Chilling broiler carcasses for 24 h in 0, 50%, and 75% vinegar water further reduced (*P* < 0.05) the pathogen cell counts (Table 1).

**Table 1:** Survival and reduction of Salmonella Typhimurium (log10 CFU/ml, Mean ± S.D.) on commercial broiler carcasses after chilling in 0 (water), 50 and 75% of vinegar water for 1 and 24 h (Laboratory studies).

Survival				Reduction	
Treatment	1h Chilling	24h Chilling		1h Chilling	24h Chilling
Control	7.31 ± 0.40cA	7.31 ± 0.40dA		—*	—*
Water	6.70 ± 0.09bB	6.22 ± 0.18cA		0.61 ± 0.09aA	1.20 ± 0.18aB
50% Vinegar	6.55 ± 0.11bB	5.14 ± 0.13bA		0.76 ± 0.11aA	2.17 ± 0.13bB
75% Vinegar	5.46 ± 0.42aB	4.79 ± 0.19aA		1.85 ± 0.42bA	2.52 ± 0.19cB

—* indicates reduction data are not available

Mean values with different letters within a column are significantly different (P<0.05)

Mean values with different capital letters within a row are significantly different (P<0.05).

For *C. jejuni*, the initial cell counts were 5.64 ± 0.37 log10 CFU/ml, chilling in 0, 50, and 75% vinegar water for 1 h reduced (*P* < 0.05) the pathogen populations (Table 2). Extending the chilling time from 1 to 24 h, the reduction slightly increased (*P* > 0.05, Table 2).

**Table 2:** Survival and reduction of Campylobacter jejuni (log10 CFU/ml, Mean ± S.D.) on commercial broiler carcasses after chilling in 0 (water), 50 and 75% of vinegar water for 1 and 24 h (Laboratory studies).

Survival				Reduction	
Treatment	1h Chilling	24h Chilling		1h Chilling	24h Chilling
Control	5.64 ± 0.37dA	5.64 ± 0.37dA		—*	—*
Water	5.42 ± 0.16cA	5.27 ± 0.08cA		0.22 ± 0.16aA	0.37 ± 0.08aA
50% Vinegar	4.18 ± 0.42bA	3.84 ± 0.53bA		1.46 ± 0.16bA	1.80 ± 0.53bB
75% Vinegar	3.64 ± 0.16aA	3.37 ± 0.61aA		2.00 ± 0.16cA	2.27 ± 0.61cA

—* indicates reduction data are not available

Mean values with different letters within a column are significantly different (P<0.05)

Mean values with different capital letters within a row are significantly different (P<0.05).

The initial cell populations of *S.* Typhimurium surrogate bacteria, *E. faecium,* on inoculated un-chilled broiler carcasses are 7.24 ± 0.12 log_10_ CFU/mL. Chilling in 0, 50 and 75% vinegar water for 1 or 24 h achieved slight reductions, resulting in increased amount of the surrogate bacteria surviving on carcasses (Table 3).

**Table 3:** Survival and reduction of surrogate bacteria Enterococcus faecium (log10 CFU/ml, Mean ± S.D.) on commercial broiler carcasses after chilling in 0 (water), 50 and 75% of vinegar water for 1 and 24 h (Laboratory studies).

Survival				Reduction	
Treatment	1h Chilling	24h Chilling		1h Chilling	24h Chilling
Control	7.24 ± 0.12bA	7.24 ± 0.12bA		—*	—*
Water	6.53 ± 0.25aA	6.78 ± 0.24aB		0.71 ± 0.25bA	0.46 ± 0.24aA
50% Vinegar	7.15 ± 0.09bB	6.66 ± 0.17aA		0.13 ± 0.05aA	0.58 ± 0.17abB
75% Vinegar	6.55 ± 0.10aA	6.57 ± 0.17aA		0.69 ± 0.10bA	0.67 ± 0.17bA

—* indicates reduction data are not available

Mean values with different letters within a column are significantly different (P<0.05)

Mean values with different capital letters within a row are significantly different (P<0.05).

### Results from MPPU pilot plant facility

The recovered *S.* Typhimurium surrogate, *E. faecium*, on inoculated but un-chilled broiler carcasses was 6.02 ± 0.29 log_10_ CFU/mL. Chilling for 24 h in water and 5 ppm of chlorine solution significantly decreased (*P* < 0.05) the surrogate bacterial counts (Table 4). Applying 2.5% lactic and citric acid blender, 50% and 75% vinegar water in the chilling tank further reduced (*P* < 0.05) the surrogate counts (Table 4).

**Table 4:** Survival and reduction of surrogate bacteria Enterococcus faecium (log10 CFU/ml, Mean ± S.D.) on mobile poultry processing unit slaughtered broiler carcasses after chilling in 0 (water), 5 ppm of chlorine, 2.5% lactic/citric acid blend, 50 and 75% of vinegar water for 24 h (Pilot plant studies).

	Survival	Reduction
Control	6.02 ± 0.29d	—*
Water	5.13 ± 0.08c	0.89 ± 0.09a
5 ppm chlorine	5.10 ± 0.16c	0.92 ± 0.18a
2.5% lactic/citric acid blend	3.45 ± 0.04a	2.57 ± 0.05b
50% Vinegar	3.64 ± 0.15ab	2.38 ± 0.17b
75% Vinegar	3.29 ± 0.17a	2.73 ± 0.18bc

—* indicates reduction data are not available

Mean values with different letters within a column are significantly different (P<0.05)

## Discussion

The laboratory portion of this study used either 0, 50, or 75% vinegar concentrations for chilling carcasses, while the MPPU portion had 5 ppm of chlorine, 2.5% of lactic and citric acid blend, and 50, 75% of vinegar water containing 2.5 and 3.75% of acetic acid were used to chill carcasses. The concentrations used in this study were well within the USDA-FSIS limits (USDA-FSIS, 2021). The 50% and 75% vinegar water used in the laboratory and pilot plant studies are based on our preliminary studies showing that less than 0.4 log reduction achieved using 4.9 to 25% vinegar. Chilling time for 1 hour is the minimal chilling time based on the average weight of the broilers used in this study. The 24-h chilling is the current standard chilling time applied in our WVU poultry processing farm. A total of 81 commercial broilers with 3 replicates (sample size n=9) were used in the laboratory test, and 72 broilers with 2 replicates (sample size n=6) were used in the MPPU study. Laboratory studies were focused on vinegar water only and the MPPU study including chlorine, lactic and citric acid blend as comparisons to vinegar, both studies included un-chilled control samples and water only chilled samples as control treatments.

Our recent study of the microbial profile of MPPU processed broiler carcasses showed that microbial cell population of carcasses tends to increase during defeathering, scalding, evisceration, and decrease following chilling, resulting in 4.05 log_10_ CFU/mL for aerobic plate counts and 3.46 log_10_ CFU/mL for generic *E. coli* (Stearns et al., 2024). The results of this study suggest that the immersion chilling process is an effective approach for inactivating foodborne pathogens on broiler carcasses, due to the extended exposure time (45 min to 24 h) of the pathogen to the chemical antimicrobial agents in the chilling tank. Chlorine was once the priority and has been well documented for its strong antimicrobial efficacy during chilling (Schambach et al., 2014). Recently, very small broiler producers in West Virginia have shown interest in understanding the antimicrobial efficacy of vinegar water during the chilling process, as they believe that vinegar water is a “clean label” antimicrobial chemical agent. In this study, the laboratory test using commercial chicken carcasses as a model verified that chilling broiler carcasses in a 50% and 75% vinegar water tank for 24 h reduced *S.* Typhimurium and *C. jejuni* by 2-2.5 and 2 log_10_ CFU/mL, respectively. The antimicrobial activity of vinegar water can be attributed to the presence of acetic acid, which is commonly present in pure commercial distilled vinegar water solutions at approximately 5%.

Acetic acid, as the main antimicrobial chemical component found in vinegar water, has been studied for its inactivation potential for poultry as a postharvest dip treatment and on carcasses during the chilling process for more than a decade. Cosansu and Ayhan (2010) found that dipping chicken legs and breasts into 1-2% of acetic acid solution for 10 min reduced *C. jejuni* by 0.78 to 1.27 and 1.27 to 1.48 log_10_ MPN/cm^2^, respectively, and the chicken leg samples treated by 2% acetic acid achieved the reduction of 3.79 log_10_ MPN/cm^2^ after 10 days stored at 4°C. Olaimat et al. (2018) immersed chicken breasts in 100 mL of washing solutions containing 5 mg/mL of acetic acid for 5 min with shaking at 150 rpm, reducing *Salmonella* (5 serovars) by approximately 2.4 log_10_ CFU/g at 4°C for 10 days of aerobic storage. Beier et al. (2019) suggested that maintaining a dissociated acetic acid concentration of 25 mM inhibits ≥ 97% of the *C. jejuni* strains isolated from broiler chicken houses in multiple states. Gonzalez-Fandos et al. (2020) also reported that dipping chicken legs at 20°C for 5 min in 1 and 2% acetic acid reduced *C. jejuni* counts by 0.80-2.01 and 1.34-2.35 log_10_ CFU/g during storage at 4°C for 9 days. A recent study by Kang et al. (2022) reported that dipping chicken skin in 0.2%, 0.6%, and 0.8% acetic acid solution for 10 min decreased *S*. Typhimurium by 0.07, 0.93, and 1.23 log_10_ CFU/cm^2^, respectively. The same study also found that 0.8% of acetic acid significantly reduced *S.* Typhimurium on the chicken breasts and drumsticks by an additional 1.35 and 1.56 log_10_ CFU/g, respectively, compared to the controls. For chilling, an early study of Dickens and Whittemore (1995) reported that applying 0.6% of acetic acid into static ice without agitation, with agitation, and in a paddle-type chiller for 1 to 3 h reduced aerobic plate counts and *Enterobacteriaceae* by 0.34 to 1.16 log_10_ and 0.50 to 1.4 log_10_ CFU/mL, respectively. The same study also found that acetic acid in the paddle chiller reduced *Salmonella* by 81.3% compared to the control with tap water only.

Previous studies on acetic acid have indicated at least a 1-2 log reduction of tested pathogens on chicken products, but the results of applying vinegar water as an antimicrobial agent have been inconsistent. Berrang et al. (2006) reported that adding 12 mL of distilled white vinegar (containing 5% acetic acid) into the colon with a battery-operated pipet pump resulted only a 1log increase (1.6 to 2.6 log_10_ CFU/sample) of *Campylobacter* before and after defeathering, which is lower than the distilled water treatment with 2.9 log increase (1.3 to 4.2 log_10_ CFU/sample). Their results suggest that applying food-grade antimicrobials to the broiler colon before scalding is effective for limiting the increase in *Campylobacter* contamination during defeathering. The most recent study of Hrustemović (2025) reported that mechanical cleaning of processing surfaces with warm water and 6% distilled vinegar decreased *Campylobacter* spp. colony counts by 3 log_10_ CFU/cm^2^ or a 42.9% reduction. The work by Hrustemović (2025) also concluded that distilled vinegar can be used as an alternative to quaternary ammonium compounds, provided that thorough mechanical cleaning of surfaces in facilities is performed. However, Henley et al. (2018) reported that chicken breasts that were washed for 10 s, 30 s, 2 min, or 5 min in 10% vinegar water reduced *Salmonella enterica* 19214 by 1.18 to 1.47 log_10_ CFU/mL, which demonstrated no differences from the tap water only treatment (1.09 to 1.35 log_10_ CFU/mL). The authors concluded that washing raw poultry in a vinegar solution is an inefficient method for removing pathogens on the chicken. This work agrees with our preliminary studies, where between 4.8 to 25% vinegar water was applied to carcasses in the lab and achieved reductions of 0.1 to 0.3 log_10_ CFU/mL of *S.* Typhimurium. The possible reason is that 4.8 to 25% commercial vinegar water only contains approximately 0.5 to 1% acetic acid, which does not reach the effective antimicrobial threshold concentration (2.5%) required to inactivate pathogens. Therefore, the greater concentrations of 50% and 75% vinegar water, containing 2.50 and 3.75% acetic acid, respectively, were used in our laboratory experimental settings and the MPPU pilot plant validation study.

Validation studies using *E. faecium* has demonstrated it to be a promising candidate for use as a *Salmonella* surrogate. These studies have been well-documented through the thermal inactivation process of a complex carbohydrate-protein matrix (Bianchini et al., 2014) and in thermally processed pet food (Ceylan and Bautista, 2015). For chicken products, our previous studies suggested that *E. faecium* demonstrated an increased heat resistance compared to *S.* Typhimurium during thermal processing of moisture-enhanced chicken breast patties (Jiang et al., 2021), salted chicken meat (Coe et al., 2025a), and mash feed samples (Coe et al., 2022; 2025b). In this study, laboratory results indicate that the reductions of *E. faecium* on broiler carcasses in the chilling tank with 50 and 75% vinegar water were lower than *S.* Typhimurium, therefore, *E. faecium* was tested in our pilot plant MPPU chilling process as a *S.* Typhimurium surrogate bacteria. The on-farm pilot plant study results showed that applying 50 and 75% of vinegar water for 24 h chilling reduced *E. faecium* by 2 log_10_ CFU/mL, which is statistically the same as the tested 2.5% lactic and citric acid blend and better than 50 ppm of chlorine water.

Future studies are needed to test the impact of vinegar water on the quality of broiler carcasses especially the color variation since acid could result in yellowing of the chicken muscles. Sensory attributes using 9 points hedonic score evaluation system with trained or customer style panels would also be important to evaluate, since applying vinegar water may cause an unacceptable to the final product. Finally, a processor safety training plan should be developed to enhance real-world applicability since an acid solution may result in corrosion over time on food contact surfaces and skin of employees.

## Conclusion

Results of this study indicated that chilling broiler carcasses in ice water containing 50 and 75% of commercial distilled vinegar water reduced *S.* Typhimurium and *C. jejuni* by 2-2.5 log *E. faecium* can be potentially used as a *Salmonella* surrogate in MPPU processing facility, and more comparison studies are needed. Future studies are needed to determine the quality and color variations of broiler carcasses chilling in vinegar water, and the state or federal level approvement is required before used in commercial settings.

## CRediT authorship contribution statement

**Carly Long:** Writing – original draft, Methodology, Investigation. **Md Shafiul Islam Rion:** Methodology, Data curation. **Corey Coe:** Methodology, Investigation. **Claire Suszynski:** Methodology, Investigation. **Reuben Adejumo:** Methodology, Investigation. **Joe Moritz:** Supervision, Methodology, Investigation. **Annette Freshour:** Supervision. **Cassandra Orndorff:** Supervision. **Timothy Boltz:** Writing – review & editing, Validation, Supervision. **Lisa Jones:** Resources. **Cangliang Shen:** Writing – review & editing, Writing – original draft, Supervision, Methodology, Investigation.

## Disclosures

The authors declare that they have no known competing financial interests or personal relationships that could have appeared to influence the work reported in this paper.
